# The impact of social distancing on mental health during the COVID-19 pandemic: a nationwide study of 4.6 million Danish adults

**DOI:** 10.1192/j.eurpsy.2025.5

**Published:** 2025-01-28

**Authors:** Andreas Geest, Barbara Bonnesen, Alexander Jordan, Louise Tønnesen, Valdemar Rømer, Charlotte S. Ulrik, Zitta Harboe, Josefin Eklöf, Pradeesh Sivapalan, Jens-Ulrik Stæhr Jensen

**Affiliations:** 1 Copenhagen Respiratory Research (COP:RESP), Department of Internal Medicine, Herlev-Gentofte University Hospital, Hellerup, Denmark; 2 Department of Respiratory Medicine, Copenhagen University Hospital – Hvidovre, Hvidovre, Denmark; 3 Department of Pulmonary and Infectious Diseases, University Hospital of Copenhagen, Copenhagen, Denmark; 4 Department of Clinical Medicine, Faculty of Health Sciences, University of Copenhagen, Copenhagen, Denmark; 5 Center for Health and Infectious Diseases Research (CHIP), University Hospital of Copenhagen, Copenhagen, Denmark

**Keywords:** COVID-19, epidemiology, mental health, pandemic, pulmonary disease

## Abstract

**Background:**

Current knowledge on psychiatric illness following periods of social distancing during the COVID-19 pandemic is mostly limited to smaller studies in selected populations. This nationwide study of all 4.6 million Danish adults examined if periods of social distancing were associated with changes in surrogate measures of mental health.

**Methods:**

All Danish adults (≥18 years) were included and rates of collection of antidepressant prescriptions, psychiatric hospital admissions, and suicide or suicide attempts for the periods March 12, 2020–May 20, 2020 (lockdown period 1), and December 21, 2020–March 1, 2021 (lockdown period 2), were compared to corresponding periods 1 year prior. Individuals were censored due to death or SARS-CoV-2 infection.

**Results:**

Antidepressant consumption increased for both period 1 and period 2, with an incidence rate ratio (IRR) of 1.02 (95% CI: 1.01–1.02, *p* < 0.001) and IRR 1.08 (95% CI: 1.08–1.09*, p* < 0.001) respectively, compared to the control periods. Psychiatric hospitalization rates decreased significantly, with an IRR of 0.65 (95% CI: 0.63–0.66, *p* < 0.001) for period 1, and IRR 0.86 (95% CI: 0.84–0.88, *p* < 0.001) for period 2. The risk of suicide did not increase in period 1, IRR 0.96 (95% CI: 0.82–1.13, *p* = 0.64), but seemed increased during period 2, IRR 1.19 (95% CI: 1.02–1.38, *p* = 0.03).

**Conclusion:**

Periods of social distancing during the COVID-19 pandemic were associated with an increase of antidepressant consumption, but decreased rates of psychiatric hospitalization. Suicide risk seemed increased during the second lockdown period.

## Introduction

It has been suggested that the unprecedented mitigation policies imposed on the public during the first and second wave of the COVID-19 pandemic could be associated with negative mental health consequences [1–3].

At the time of the initial outbreak, no approved vaccines or curative treatments existed; thus, the containment of the pandemic relied on non-pharmaceutical measures, leading to nationwide implementation of social distancing measures. The severity of mitigation policies varied during the pandemic with the periods March 12, 2020–May 20, 2020 (lockdown period 1), and December 21, 2020–March 1, 2021 (lockdown period 2), being the most heavily impacted. Measures imposed to ensure social distancing included bans on private gatherings of more than 10 people and closing of schools and liberal professions [4] (see Supplementary Material *, Danish mitigation strategies*, for a comprehensive overview of Danish mitigation strategies during COIVD-19).

Prolonged periods of social distancing can cause isolation, where social connections and interactions are absent or severely hampered [5]. Social isolation can, depending on individual differences, lead to loneliness, an independent but often co-occurring construct. Loneliness is a subjective feeling of distress that can occur when social interactions are perceived as inadequate. The individual perception of decreased social interaction thus facilitates the link between loneliness and social isolation [5]. Loneliness is associated with suicidal ideation and symptoms related to mental health [5, 6]. Several studies have suggested that the COVID-19 pandemic and the subsequent changes in social interactions have impacted the mental health status of the general population [2, 3].

Social distancing measures served as pivotal tools in pandemic control and proved effective in stemming the transmission of disease during the COVID-19 pandemic [7]. With the potential of similar pandemics in the future, it is likely that the implementation of social distancing yet again will become an important tool for disease mitigation; thus, it is imperative to gain a better understanding of the related mental health effects [8–10].

This nationwide cohort study of the entire adult population of Denmark investigated the potential impact of severe social distancing measures on mental health outcomes. Specifically, we aimed to assess whether these measures were associated with mental health disorders as assessed by prescription of antidepressants, psychiatric hospitalization, and cases of suicide or suicide attempt. We hypothesized that social distancing was associated with an increased risk of collection of prescriptions of antidepressants, admission to a psychiatric hospital department, and suicide including suicide attempts in Denmark.

## Methods

This is a nationwide retrospective population-based study utilizing the National Danish registries. The study was approved by the Danish Data Protection Agency (j.no. P-2021-360). Informed consent for retrospective studies is not required in Denmark. All Danish citizens are linked to a unique identification number in the Civil Registration System (CRS) [11], which, in this study, was used for exact linkage at an individual level between registers, ensuring complete follow-up.

### Exposure periods

**Figure 1.**
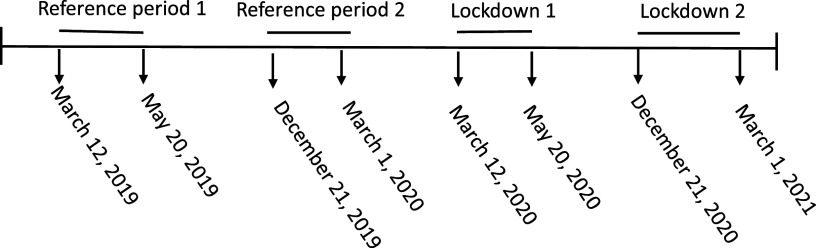
Definition of study periods.

There were two lockdown periods, and thus exposure periods, during the pandemic in Denmark: March 12, 2020–May 20, 2020 (lockdown period 1), and December 21, 2020–March 1, 2021 (lockdown period 2), with corresponding reference periods: March 12, 2019–May 20, 2019 (reference period 1), and December 21, 2019–March 1, 2020 (reference period 2) (Figure 1).

### Data sources

- The Danish CRS includes individual information on the unique personal identification number, name, sex, date of birth, and vital status [11].
- The Danish National Patient Registry holds information on all admissions to Danish hospitals since 1977 and hospital outpatient clinic visits since 1995. Each hospital visit is coded by physicians with one primary diagnosis and one or more secondary diagnoses, according to the International Classification of Diseases, eighth revision codes until 1994 and ICD-10 thereafter [12].
- The Danish National Health Service Prescription Database holds information on all prescriptions that have been dispensed in Danish pharmacies, since 2004 (coded according to the ATC classification system), including the following information in terms of OCS: the date of dispensation, the quantity dispensed as well as the strength and formulation of all prescriptions that have been dispensed from Danish Pharmacies. All pharmacies are required by Danish legislation to provide information that ensures complete and accurate registration [13].
- The Cause of Death Register (DAR) holds information on all registered causes of deaths of Danish citizens since 1970 [14].
- The Danish Microbiology Database containing data on PCR-confirmed SARS-CoV-2 infection since February 2020 [15].

### Study population

The study population included all Danish adults (≥18 years) residing in Denmark (not including Greenland and Faroe Islands) as of January 1, 2019, and throughout the study period until March 1, 2021. No exclusion criteria were applied. Individuals were censored in case of death or SARS-CoV-2 infection. The latter was based on SARS-CoV-2 PCR tests collected from nationwide microbiological laboratories.

### Outcomes

All outcomes were quantified during lockdown periods 1 and 2, as well as during reference periods 1 and 2 as described in “exposure periods.”

The primary outcome was the collection of a prescription for antidepressants (Anatomical Therapeutic Chemical classification codes, ATC, N06A including all subgroups). Antidepressant prescription collection was considered a binary variable with two possible outcomes, either none or at least one prescription.

The two secondary outcomes were (1) admissions to a psychiatric ward and (2) suicide or suicide attempt. A psychiatric admission was defined as any psychiatric ward contact lasting a minimum of 24 h, with a primary diagnosis of either depression (ICD-10: DF32, DF33, DF34); anxiety (ICD-10: DF40-42, DF48, DF50); or bipolar disorder (ICD-10 codes DF30-31), including maniac episodes (ICD-10: DF30).

Suicide was defined as “dead” in the CRS and cause of death in the Cause of Death Register as serious self-harm or poisoning from mild pain relievers, including paracetamol (ICD-10 DT39). Suicide attempt was defined as a hospital contact registered with a primary diagnosis of serious self-harm or poisoning from mild pain relievers, including paracetamol (ICD-10 DT39).

A post hoc sensitivity analysis was performed on suicide data across a combined exposure period (lockdown period 1 and lockdown period 2) due to low amounts of suicide and suicide attempts observed in the main analysis.

Additional post hoc sub-analyses were made on all endpoints stratifying for age and gender (see Supplementary Figures 3–5). Antidepressant consumption was also stratified into groups of de novo prescriptions (no prior prescription of antidepressants within 12 months of the particular period, lockdown, or reference) and non-de novo prescriptions (at least one prescription of any antidepressant within 12 months of the particular period, lockdown, or reference) (see Supplementary Table 1). Similarly, the endpoint regarding psychiatric hospitalization was stratified for de novo admissions and readmissions (at least one psychiatric admission of a minimum of 24 h within 12 months of the particular period, lockdown, or reference) (see Supplementary Table 1).

To investigate the stockpiling of drugs at patients’ homes and the potential impact on the collection of antidepressant prescriptions, an analysis of the usage of enalapril, as a control drug, was conducted. Enalapril is widely used to treat chronic conditions such as hypertension and heart failure; thus, the pandemic is not expected to have any major immediate impacts on its consumption, therefore making it an ideal control drug for investigating stockpiling (see Supplementary Table 2).

### Statistical analysis

Categorical variables were presented as frequencies and absolute numbers. Continuous variables were presented as means with 95% confidence intervals or median values with interquartile ranges depending on the data distribution. Primary and secondary outcomes were presented as incidence rates (IRs) and IR ratios (IRRs) with corresponding 95% confidence intervals, and were calculated and compared using two-sided *t*-statistics. R software was used for statistical analysis.

## Results

**Figure 2.**
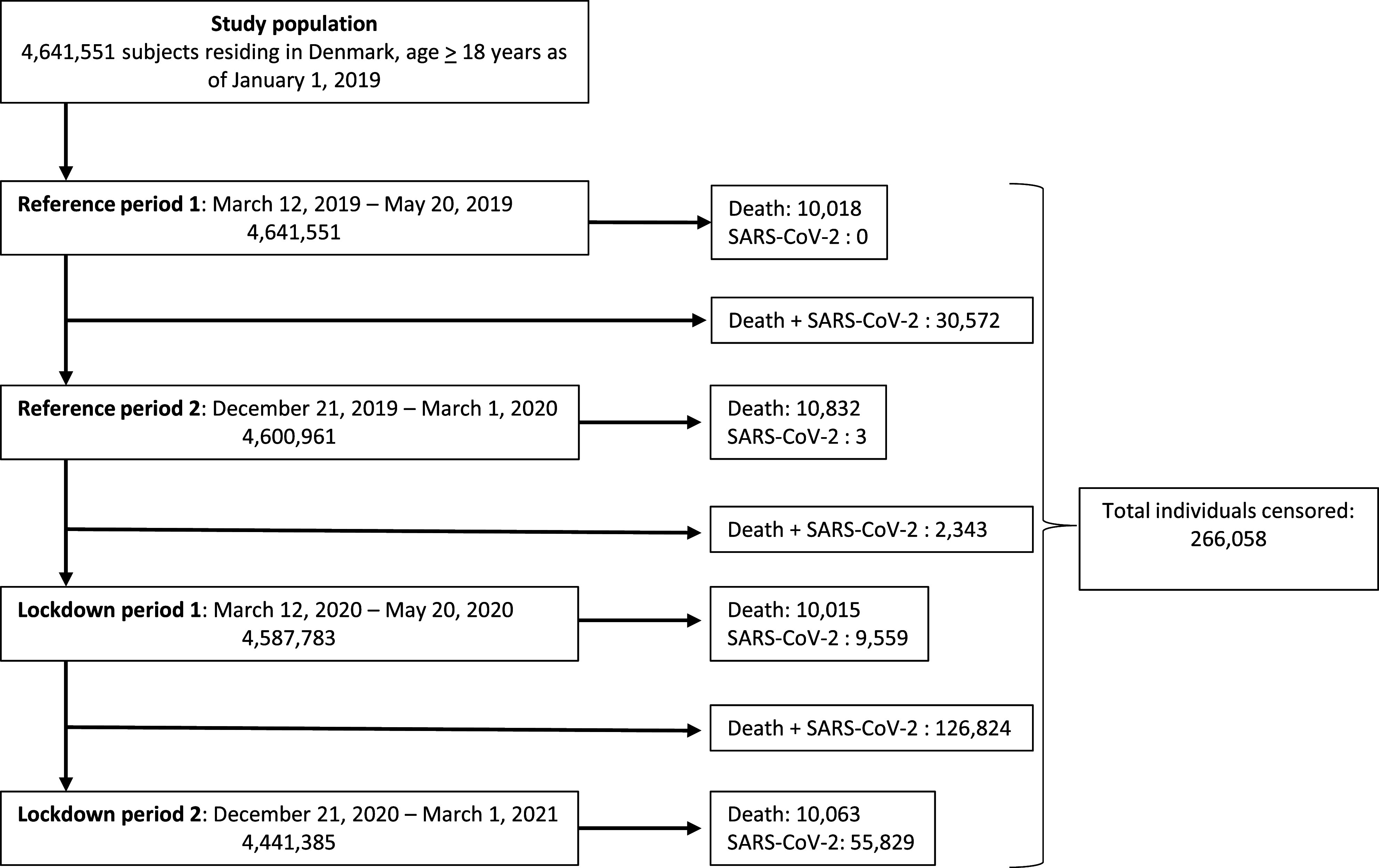
Study flowchart. All adults (>18 years) residing in Denmark were included. No exclusion criteria were defined. Subjects were censored due to death or SARS-CoV-2 infection.

We identified a total of 4,641,551 individuals aged > 18 years (Figure 2). Baseline demographics and clinical characteristics are summarized in Table 1. Of these individuals, 595,175 (12.8%) had received at least one prescription of psychoactive medication and 231,847 (5.0%) suffered from a specialist-treated psychiatric illness. As seen in Table 1, the baseline demographics remain similar during all four periods, with a slight decrease in median age and comorbidity score. This is primarily due to individuals censored for death being older and having more comorbidities than the average population, thus slightly altering the demographics during the study period. The censoring for death was consistent throughout all four periods, varying from 10,015 to 10,832 deaths per period.

**Table 1.** Baseline patient demographic and clinical characteristics in a population of adult Danish citizens ≥ 18 years by January 1, 2019

Characteristics	Period 1^a^		Period 2^b^	
	Reference period (*N* = 4,641,551)	Lockdown period (*N* = 4,587,783)	Reference period (*N* = 4,600,961)	Lockdown period (*N* = 4,441,385)
Age, median (IQR)	49 (33–64)	48 (33–64)	49 (33–64)	48 (33–63)
Male sex, *n* (%)	2,287,750 (49.29)	2,260,230 (49.27)	2,267,067 (49.27)	2,188,263 (49.27)
Medication				
Any psychoactive medication, *n* (%)	595,175 (12.82)	574,085 (12.51)	578,776 (12.58)	549,644 (12.38)
*Antidepressants*	393,051 (8.47)	379,593 (8.27)	382,618 (8.32)	363,477 (8.18)
*BZD and BZD-like*	244,572 (5.27)	233,651 (5.09)	236,046 (5.13)	222,686 (5.01)
*Antipsychotics*	113,364 (2.44)	108,868 (2.37)	109,803 (2.39)	104,232 (2.35)
*Lithium*	8733 (0.19)	8,564 (0.19)	8,605 (0.19)	8,269 (0.19)
Comorbidities				
Specialist-treated psychiatric illness, *n* (%)	231,847 (5.00)	227,353 (4.96)	228,359 (4.96)	219,555 (4.94)
*Depression*	85475 (1.84)	82,959 (1.81)	83,502 (1.81)	79,470 (1.39)
*Anxiety disorders*	64,706 (1.39)	63,762 (1.39)	63,988 (1.39)	61,812 (1.39)
*Schizophrenia*	26,510 (0.57)	26,052 (0.57)	26,159 (0.57)	25,389 (0.57)
*Bipolar*	15,266 (0.33)	14,946 (0.33)	15,019 (0.33)	14,434 (0.32)
COPD, *n* (%)	144,288 (3.11)	136,234 (2.97)	138,058 (3.00)	127,844 (2.88)
Diabetes mellitus, *n* (%)	138,467 (2.98)	131,607 (2.87)	133,115 (2.89)	123,997 (2.79)
Stroke and transient cerebral ischemia, *n* (%)	87,699 (1.89)	82,235 (1.79)	83,448 (1.81)	77,227 (1.74)
Charlson comorbidity index, mean (CI)	1.14 (1.14–1.14)	1.11 (1.11–1.11)	1.12 (1.12–1.12)	1.10 (1.09–1.10)

Abbreviations: IQR, interquartile range; COPD, chronic obstructive lung disease; CI, 95% confidence interval; BZD, benzodiazepine.

a Lock down period 1: March 12, 2020–May 20, 2020 (reference period 1: March 12, 20219–May 20, 2019).

b Lockdown period 2: December 21, 2020–March 1, 2021 (reference period 2: December 21, 2019–March 1, 2020).

The IRs of collection of antidepressant prescriptions during reference period 1 and lockdown period 1 were 564 per 100,000 person-weeks and 574 per 100,000 person-weeks, respectively. During reference period 2 and lockdown period 2, the IR was 552 per 100,000 person-weeks and 598 per 100,000 person-weeks, respectively (see Table 2). This corresponds to an IRR of 1.02 (95% CI: 1.01–1.02, *p* < 0.001) for lockdown period 1 and 1.08 (95% CI: 1.08–1.09, *p* < 0.001) for lockdown period 2. Cumulative incidences of collections of antidepressant prescriptions are illustrated in Supplementary Figures 1 and 2.

**Table 2.** Weekly incidences of psychiatric outcomes in periods with implemented COVID-19 lockdown measures compared to reference periods (same dates 1 year before) in a population of adult Danish citizens ≥ 18 years

Outcomes	Period 1^a^		Period 2^b^	
	Reference period(*N* = 4,641,551)	Lockdown period(*N* = 4,587,783)	Reference period(*N* = 4,600,961)	Lockdown period(*N* = 4,441,385)
Antidepressants				
IR	564 per 100,000	574 per 100,000	552 per 100,000	598 per 100,000
IRR (95% CI)	Ref.	1.02 (1.01 to 1.02) *p* < 0.001	Ref.	1.08 (1.08 to 1.09) *p* < 0.001
Psychiatric hospital admission				
IR	36.96 per 100,000	23.85 per 100,000	35.93 per 100,000	30.90 per 100,000
IRR (95% CI)	Ref.	0.65 (0.63 to 0.66) *p* < 0.001	Ref.	0.86 (0.84 to 0.88) *p* < 0.001
Suicide and suicide attempt				
IR	0.64 per 100,000	0.62 per 100,000	0.69 per 100,000	0.82 per 100,000
IRR (95% CI)	Ref.	0.96 (0.82 to 1.13) *p* = 0.64	Ref.	1.19 (1.02 to 1.38) *p* = 0.03

*Note*: Persons/Subjects were censored when dead or PCR-confirmed SARS-CoV-2 infection.

Abbreviations: IR, incidence rate; IRR, incidence rate ratio; CI, confidence interval.

a Lockdown period 1: March 12, 2020–May 20, 2020 (reference period 1: March 12, 20219–May 20.

b Lockdown period 2: December 21, 2020–March 1, 2021 (reference period 2: December 21, 2019–March 1, 2020).

The IRs of psychiatric hospitalization during reference period 1 and lockdown period 1 were 36.9 per 100,000 person-weeks and 23.9 per 100,000 person-weeks, respectively. During reference period 2 and lockdown period 2, the IR was 35.9 per 100,000 and 30.9 per 100,000, respectively. This corresponds to an IRR of 0.65 (95% CI: 0.63–0.66, *p* < 0.001) for lockdown period 1 and 0.86 (95% CI: 0.84–0.88, *p* < 0.001) for lockdown period 2 (Table 2). Thus, social distancing was associated with a significantly decreased risk of psychiatric hospitalization during both lockdown periods, particularly during the first period.

For suicide and suicide attempts, no statistically significant difference was found between reference period 1 and lockdown period 1. However, during the second period of lockdown, the IR was found to increase from 0.69 per 100,000 during reference period 2 to 0.82 per 100,000 during lockdown period 2, corresponding to an IRR of 1.19 (95% CI: 1.02–1.38, *p* < 0.03) (Table 2).

### Post hoc sub-analyses

From sub-analyses stratifying for both age and gender, we saw the biggest rise in antidepressant consumption for the youngest age group (18–32 years), with an IRR of 1.11 (95% CI: 1.09–1.14, *p* < 0.001) for women and 1.09 (95% CI: 1.06–1.12, *p* < 0.001) for men during lockdown period 1, and with similar trends in lockdown 2, IRR 1.23 (95% CI: 1.21–1.25, *p* < 0.001) for women and 1.18 (95% CI: 1.15–1.21, *p* < 0.001) for men (Supplementary Figure 3).

For psychiatric hospital admissions, the impact of the lockdown periods was most apparent among the elderly, age > 63 years, IRR 0.51 (95% CI: 0.47–0.56, *p* < 0.001) for women and 0.60 (95% CI: 0.53–0.68, *p* < 0.001) for men during lockdown period 1 compared to 0.72 (95% CI: 0.66–0.78, *p* < 0.001) for women and 0.81 (95% CI: 0.73–0.91, *p* < 0.001) for men during lockdown period 2 (Supplementary Figure 4).

For suicide and suicide attempts, no significant results were found during lockdown 1 when stratifying for age and gender. However, during lockdown period 2, the IRR was increased for men above 63 years, IRR 3.04 (95% CI: 1.72–5.38, *p* < 0.001), as well as for women above 63 years, IRR 1.63 (95% CI: 1.02–2.60, *p* = 0.041) (Supplementary Figure 5).

During lockdown period 1, there was an increased rate of antidepressant prescriptions with current users as the rate decreased for de novo prescriptions. For lockdown period 2, we saw increases within both groups, with the biggest increase in de novo prescriptions (Supplementary Table 1).

For psychiatric hospitalization, the decrease was most pronounced for those who had a previous hospitalization within 12 months compared to the group with no previous psychiatric admission (Supplementary Table 1).

No increase was seen in the consumption of the “control drug” (enalapril) during either lockdown period 1 (Supplementary Table 2).

The post hoc sensitivity analysis on combined suicide and suicide attempts showed no significant change in events from combined reference (IR: 0.66) to combined exposure periods (IR: 0.71), IRR 1.07 (95% CI: 0.96–1.20, *p* = 0.20).

## Discussion

In this nationwide registry-based cohort study of 4.6 million Danish inhabitants, with a follow-up time of 3.4 million person-years for the primary outcome, we found that the periods of social distancing implemented to mitigate the COVID-19 pandemic were associated with an increase in collected prescriptions of antidepressant medication along with a significantly lower admission rate to psychiatric wards compared to the pre-pandemic reference periods. No significant difference was detected in rates of suicide and suicide attempts during the initial lockdown period or in the post hoc combined exposure analysis. However, suicide risk seemed to increase in the second lockdown period. Post hoc sub-analyses showed that the increased suicide risk was most pronounced among the elderly. It is important to interpret the findings regarding suicide cautiously, considering the limited statistical power. Correspondingly, a systematic review of pre- and peri-pandemic suicide data across 13 databases found a nonsignificant downward trend for suicide rates during the pandemic; however, the study showed increasing trends for both suicidal ideation and suicide attempts during the pandemic [3].

The prescription of antidepressants exhibited a more substantial rise during lockdown period 2 compared to the increase observed in lockdown period 1. This trend might be related to fatigue experienced by individuals due to the prolonged impact of the pandemic [16]. Factors such as prolonged social isolation, economic challenges, and general uncertainties about the future could have potentiated the negative mental health effects of social distancing, potentially leading to increased antidepressant consumption during the later stages of the pandemic. An impact was seen across all age groups and genders; however, post hoc sub-analyses showed that impacts were most pronounced amongst younger individuals between 18 and 32 years old. This age distribution corresponds well with other studies on mental health during COVID-19 [17]. Similar increases were found for current users of antidepressants during both lockdown periods, whereas the number of new users decreased during the first lockdown but increased during the second.

A Swedish study of 1.4 million inhabitants in the region of Scania found no changes in the trends of common psychotropic medications after March 2020, concluding that public mental health was not affected by the COVID-19 pandemic in a way that altered the use of psychotropic medication. The Swedish government’s strategies for mitigating the COVID-19 pandemic differed from those applied in Denmark and most other countries, relying primarily on recommendations rather than restrictions, thus abstaining from full-scale lockdown [18]. As there are otherwise noteworthy similarities between the two Scandinavian populations, the increased consumption of antidepressants found in this current study, compared to that of the Swedish study, could be attributed to the more extensive social distancing measures applied in Denmark compared to Sweden. However, it is important to note that mobility data show similar trends for cell phone mobility data from April 2020 and onward when comparing Sweden and other Nordic countries, including Denmark [19]. This suggests that differences in real-world pandemic mitigation strategies are more subtle than otherwise indicated by steps taken at a national level.

An analysis conducted by the Danish Health Data Authority concludes that the Danish consumption of antidepressants in 2020 has been stable in relation to the last 5 years; however, similar to the findings of this current study, they found an increased consumption in March 2020 and December 2020, corresponding to the initiation of the first and second national lockdown periods [20].

No increases in enalapril usage were seen during either lockdown period and, thus, there is no clear evidence of stockpiling of medication occurring and subsequently affecting the findings of this study

The increased consumption of antidepressants contrasts with the decreased psychiatric hospitalization rate. This could, however, be attributed to an elevated threshold for healthcare contact during the pandemic rather than a lower prevalence of psychiatric disorders requiring hospitalization. Somatic diseases, such as cardiovascular disorders, saw similar lower incidences in Denmark during the COVID-19 pandemic [21, 22]. The studies in question suggest that lower admission rates are, in part, caused by a crisis-driven threshold raise for patients contacting a physician when experiencing symptoms and for the physician agreeing to a consultation. Similar mechanisms can explain the decreased psychiatric hospitalization rate, potentially unveiling a temporary underdiagnosis of psychiatric (as well as somatic) disorders, with issues related to untreated mental illness presenting themselves at a later stage.

The increased consumption of antidepressants could, in part, be explained by a shift from inpatient care to outpatient care, highlighted by the decreased rate of psychiatric hospitalization. During lockdown period 2, however, the absolute increase in the number of people collecting a prescription for antidepressants greatly exceeds the corresponding decrease in psychiatric hospitalization. The decrease in inpatient care can therefore only explain a small part of the increased consumption of antidepressants.

A major strength of this study is that we followed the entire adult Danish population, allowing for a sample size of 4.6 million Danish inhabitants and providing extensive statistical power. The inclusion of essentially all Danish adult residents in the cohort allows generalizability to national populations in contrast to other studies based on smaller, selected databases, which may not be representative of the general population. Secondly, this study was able to compare virtually the same population with itself at different points in time, with subtle differences in the actual populations, thereby limiting the effects of potential confounders to some extent.

Thirdly, due to the extensiveness of the Danish registries on health data, no subjects were lost to follow-up. We had access to complete and validated data on prescriptions, hospital admissions, and causes of death.

Furthermore, the censoring of SARS-CoV-2-infected individuals was based on a PCR-validated SARS-CoV-2 diagnosis via real-time nationwide microbiological data from central laboratories and no self-tests. COVID-19 infection has been linked to an increased use of psychoactive medication [23] and could contribute to an increased signal, unrelated to the social distancing measures; thus, SARS-CoV-2-infected individuals were censored. This does, however, also introduce a slight risk of bias, as those infected with COVID-19 differ from the total population as they are generally younger, less medicated with psychoactive medication, and have fewer comorbidities [23]. However, this amounts to <200,000 individuals censored due to SARS-CoV-2 infection, out of a total sample size of 4.6 million, and is therefore not expected to drive a signal.

There are some limitations to this study. This study holds no information on adherence to social distancing guidelines and thus solely relies on governmental implementations. A lack of adherence would tend to weaken the signal.

It has been shown from survey data that living alone during COVID-19 was associated with higher levels of loneliness and lower life satisfaction [24]. This study does not have access to data on the type of residence and dwelling; this would otherwise have added valuable information on whether specific living situations (i.e. living alone vs. other living situations), would impact the endpoints investigated in this study.

The analyses of this study were based on observations before and after the intervention of social distancing; thus, the follow-up was limited to the exposure time. The findings of this study would be further strengthened by observing an expected normalization of both antidepressant consumption and psychiatric hospitalization in the corresponding time periods following the removal of social distancing measures. The collection of antidepressant medications does not necessarily reflect the mental health status of the population, as filed prescriptions are also influenced by several other factors. Other psychoactive medications can, too, be used to reflect the mental health status of a population. However, depression and anxiety disorders account for more than half the specialist-treated psychiatric illnesses within the study population. For both conditions, antidepressants are often the first-line pharmacological treatment. In the current study, antidepressants account for two-thirds of the total use of psychoactive medication in the population. Furthermore, due to frequent reports of symptoms related to anxiety and affective disorders during the COVID-19 pandemic, it is hypothesized that these conditions are the psychiatric disorders most likely to be influenced by the lockdown periods [25]. Therefore, we believe that the consumption rate of antidepressant medication is a reliable indicator of public mental health status during the COVID-19 lockdowns, but recognize that it does not provide a complete picture.

The impacts of the lockdowns are complex and several factors are likely to have influenced the mental health of the public during the pandemic; these include anxiety toward the future, job and economic uncertainties, governmental distrust, and fear of dying or losing loved ones. It has also been hypothesized that the lockdown periods have had positive impacts, such as increased time spent with family and being outdoors, along with a deceleration of the societal rhythm.

With data based on a nationwide cohort, this study aimed to provide valid and generalizable results without nonresponse-induced bias. To our knowledge, this study is currently the largest study of nationwide data on the consumption of antidepressant medication, psychiatric hospitalization, and suicide and suicide attempts.

In conclusion, in this nationwide cohort study of the entire 4.6 million adult population of Denmark, we found an increase in the consumption of antidepressant medication, in particular, amongst young adults during two separate periods of social distancing during the COVID-19 pandemic. Concurrently, we saw a significantly decreased rate of psychiatric hospitalization. Rates of suicide and suicide attempts increased during the second lockdown period, especially among the elderly.

The results of this study should contribute to the debate over an increased monitoring of possible residual damage to public mental health following the COVID-19 pandemic. Simultaneously, this study brings valuable insights into the possible effects of social distancing, which can and should be taken into consideration by governments and healthcare authorities in the event of a future pandemic demanding a social distancing-based mitigation strategy.

## Supporting information

Geest et al. supplementary material Geest et al. supplementary material

## Data Availability

We believe that knowledge sharing increases the quantity and quality of scientific results. Sharing of relevant data will be discussed within the study group upon reasonable request. All data sharing should respect Danish legislation.

## Supplementary material

The supplementary material for this article can be found at http://doi.org/10.1192/j.eurpsy.2025.5.
