# Supplementary material for: The impact of social distancing on mental health during the COVID-19 pandemic: a nationwide study of 4.6 million Danish adults
Source: Eur Psychiatry. 2025 Jan 28;68(1):e30. doi: 10.1192/j.eurpsy.2025.5 (PMC11883780; doi:10.1192/j.eurpsy.2025.5)
Supplement: Geest et al. supplementary material [file S0924933825000057sup001.docx]

# Supplementary material

*Overview: Danish mitigation policies during SARS-CoV-2* *pandemic* [4]

Following the initial outbreak in Denmark March 2020, the general guidelines issued by the Danish government were to ensure good hand hygiene, good coughing etiquette, avoiding physical contact and ensuring a distance of 2 meters to others. As a response to an acceleration in the spread of Sars-CoV-2, community-level social distancing was implemented on March 13, 2020, to reduce and mitigate the infection. These implementations meant closing of public youth education and other educational institutes, closing of public cultural institutions, such as libraries, and that all public employees performing non-critical functions were send home. During the following days, this lockdown was extended to include closing of restaurants, café’s, nightlife etc. and a ban on private gatherings of more than 10 people was issued.

Following a stabilization of the initial outbreak and a decrease in the number of people infected with Sars-Cov-2, a multi-staged reopening plan was presented by the Danish government, with the first stage including reopening of day care centers, public schools, and some youth education on April 15, 2020. The initial reopening was followed by reopening of liberal professions on April 20, 2020. A continued decrease in the number of registered SARS-CoV-2 cases allowed reopening of restaurants, bars, cafe’s etc. and a return to normal school attendance for students in public schools on May 18, 2020.

A complete reopening of society was postponed due to an increase in the number of people infected in August 2020 and restrictions were later re-imposed to combat a second wave of SARS-CoV-2 infections in Denmark. This second wave led to government issued restrictions, similar to those of the first wave, in which public schools were closed along with all non-essential stores by December 25, 2020. A decrease in the number of SARS-CoV-2 infections along with the inception of a national vaccination program against SARS-CoV-2, meant that a partial reopening was initiated by March 1st, 2021.


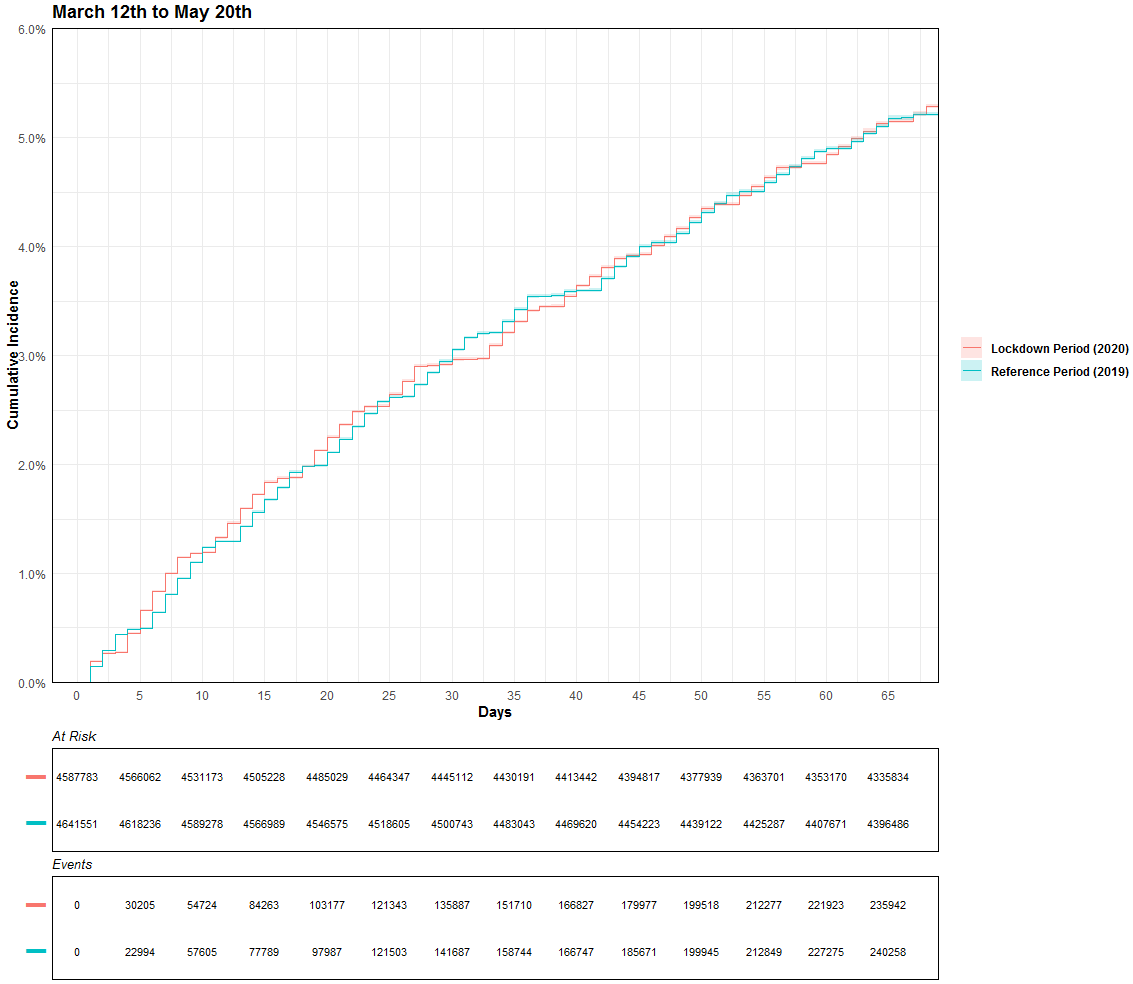


Supplementary figure 1: Cumulative incidences of collections of prescriptions for antidepressive medication for Lock down period 1: March 12, 2020 – May 20, 2020, and reference period 1: March 12, 20219 – May 20, 2019.


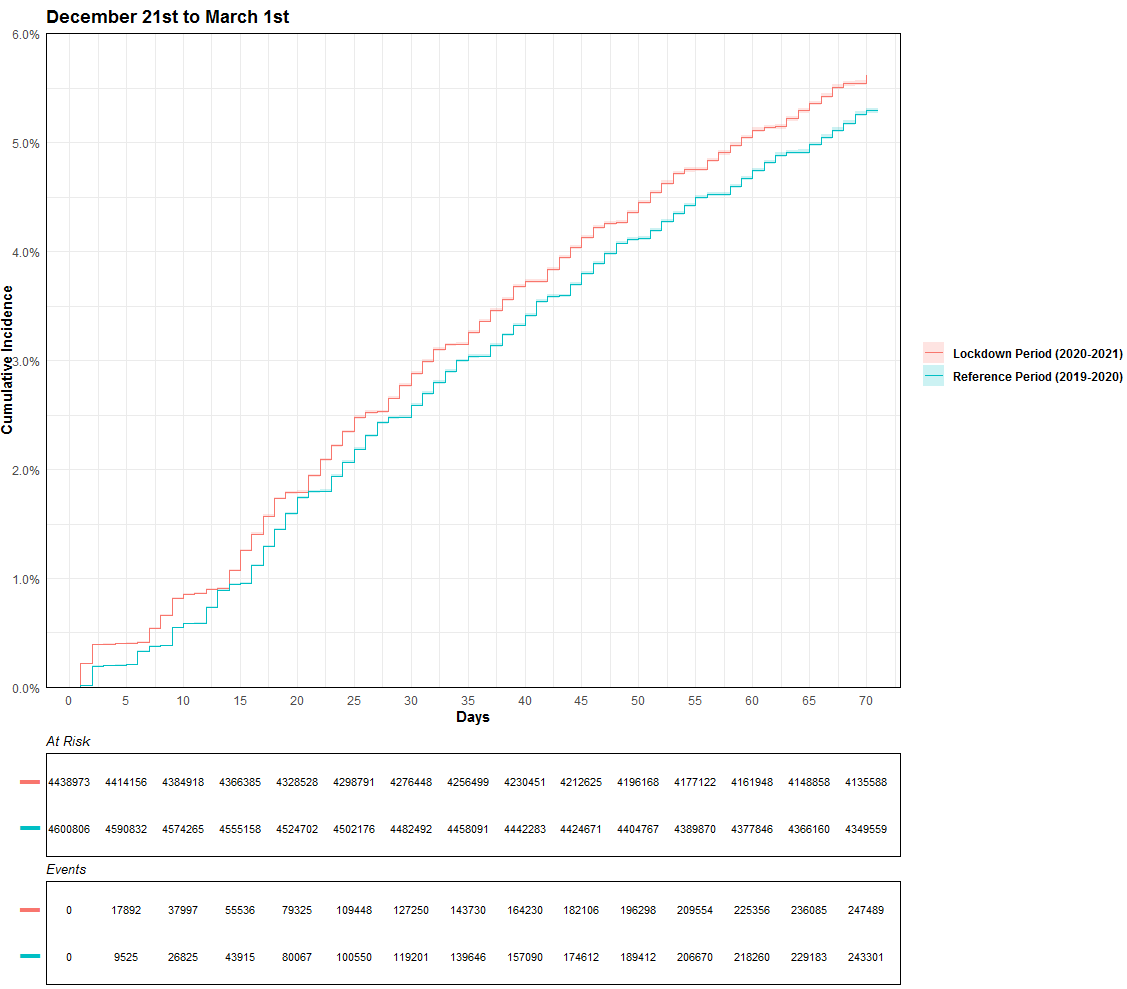


Supplementary figure 2: Cumulative incidences of collections of prescriptions for antidepressive medication for Lock down period 2: December 21, 2020 – March 1, 2021 and reference period 2 : December 21, 2019 – March 1, 2020.


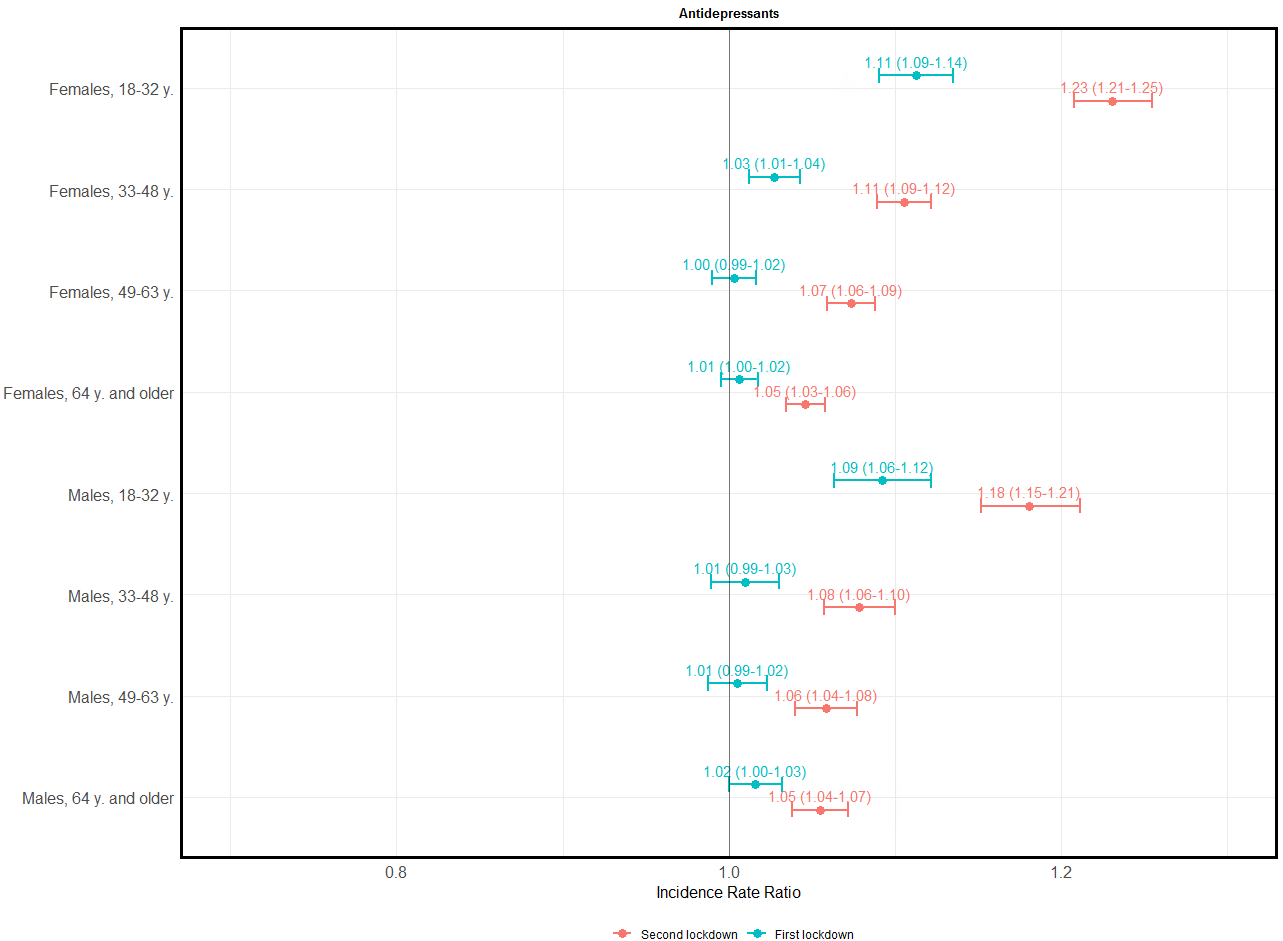


Supplementary figure 3: Subanalyses stratified by age and gender. Forrest plot showcasing incidence rate ratios for the collection of prescriptions of antidepressant medication during lockdown 1 (blue) and lockdown 2 (red) in relation to corresponding reference periods 1 year prior.

Supplementary figure 4: Subanalyses stratified by age and gender. Forrest plot showcasing incidence rate ratios of psychiatric hospitalizations during lockdown 1 (blue) and lockdown 2 (red) in relation to corresponding reference periods 1 year prior


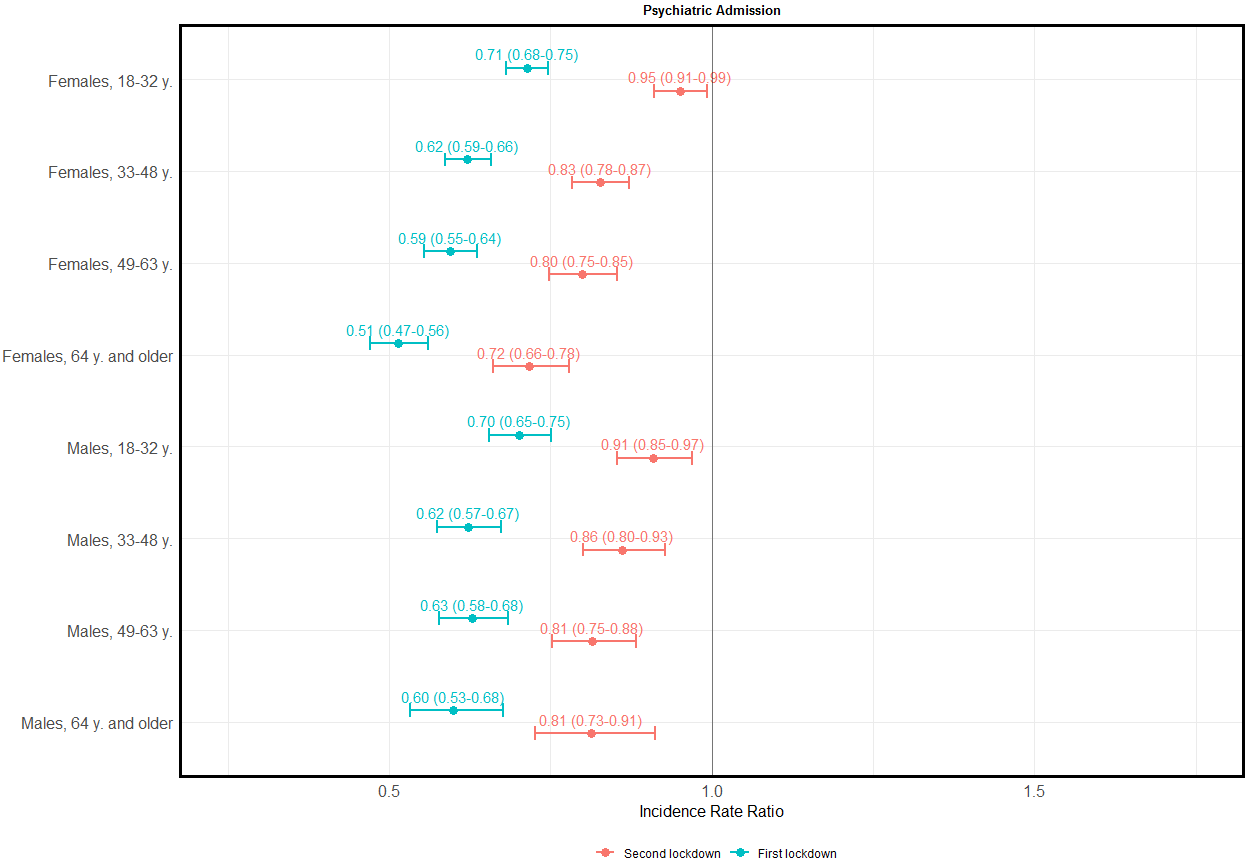


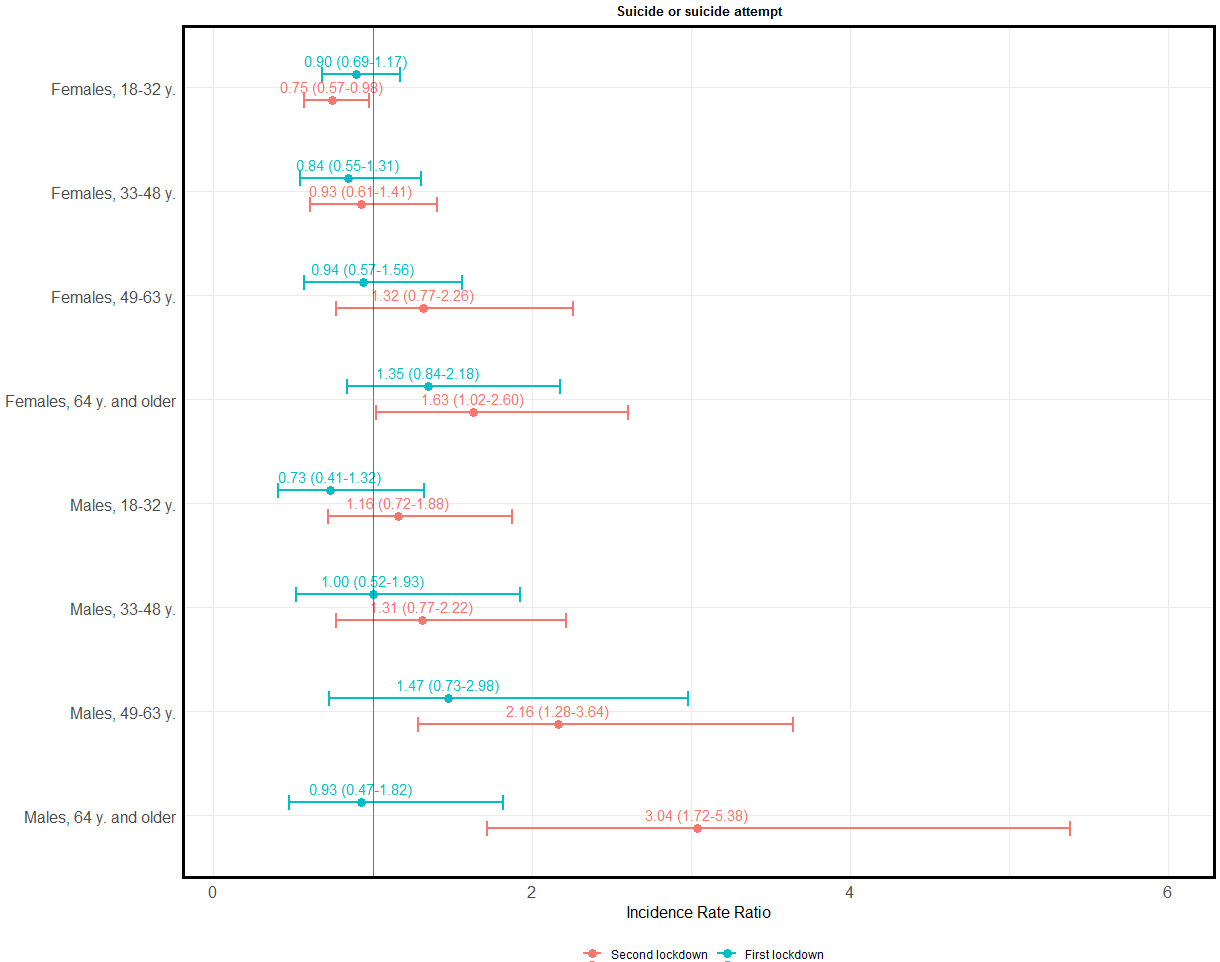


Supplementary figure 5: Subanalyses stratified by age and gender. Forrest plot showcasing incidence rate ratios of suicides and suicide attempts during lockdown 1 (blue) and lockdown 2 (red) in relation to corresponding reference periods 1 year prior

| **Outcomes** | ***Period 1 **** | | ***Period 2 ***** | |
| --- | --- | --- | --- | --- |
|  | **Reference Period**  (N = 4,641,551) | **Lockdown Period**  (N = 4,587,783) | **Reference Period**  (N = 4,600,961) | **Lockdown Period**  (N = 4,441,385) |
| **Antidepressants (de novo)** |  |  |  |  |
| IRR (95% CI) | Ref. | 1.04 (1.04 ; 1.05)   *p < 0.001* | Ref. | 1.05 (1.05 ; 1.05)  p < 0.001 |
| **Antidepressants (current users)** |  |  |  |  |
| IRR (95% CI) | Ref. | 0.83 (0.82 ; 0.84).  *p <* 0.001 | Ref. | 1.11 (1.10 ; 1.12)  *p* < 0.001 |
| **Psychiatric hospital admission (de novo)** |  |  |  |  |
| IRR (95% CI) | Ref. | 0.35 (0.33 ; 0.36).  *p <* 0.001 | Ref. | 0.90 (0.86 ; 0.95)  *p* < 0.001 |
| **Psychiatric hospital admission**  **(re-admissions)** |  |  |  |  |
| IRR (95% CI) | Ref. | 0.22 (0.21 ; 0.22)   *p < 0.001* | Ref. | 0.77 (0.75 ; 0.79)  p < 0.001 |
| Abbreviations: IR, Incidence Rate; IRR, Incidence Rate Ratio; CI, Confidence Interval.  *Lock down period 1: March 12, 2020 – May 20, 2020 (reference period 1: March 12, 20219 – May 20, 2019)  ** Lock down period 2: December 21, 2020 – March 1, 2021 (reference period 2: December 21, 2019 – March 1, 2020) | | | | |

Supplementary table 1: de novo versus non-de novo antidepressant consumption and psychiatric admissions. De novo prescriptions were defined as: no prior prescription of antidepressants within 12 months of the particular period, lockdown or reference. De novo psychiatric admissions were defined as: at least 1 psychiatric admission of minimum 24 hours within 12 months of the particular period, lockdown or reference.

| \| **Outcomes** \| **Lockdown 1** \| **Lockdown 2** \| \| --- \| --- \| --- \| \| **Enalapril (control drug)** \|  \|  \| \| IRR (95% CI) \| 0.96 (0.95 ; 0.97) *p < 0.001* \| 1.01 (1.00 ; 1.02)   *p = 0.07* \|   Supplementary table 2: incidence rate ratios for the collection of prescriptions for Enalapril during lockdown 1 and lockdown 2 in relation to corresponding reference periods. |  |  |  |  |
| --- | --- | --- | --- | --- | --- | --- | --- | --- | --- | --- | --- | --- | --- |
